# Tracking Transplanted Bone Marrow Stem Cells and Their Effects in the Rat MCAO Stroke Model

**DOI:** 10.1371/journal.pone.0060049

**Published:** 2013-03-29

**Authors:** Gregory V. Goldmacher, Rena Nasser, Daniel Y. Lee, Sargon Yigit, Robert Rosenwasser, Lorraine Iacovitti

**Affiliations:** 1 Farber Institute for Neurosciences, Thomas Jefferson University, Philadelphia, Pennsylvania, United States of America; 2 Department of Neuroscience, Thomas Jefferson University, Philadelphia, Pennsylvania, United States of America; 3 Department of Neurological Surgery, Thomas Jefferson University, Philadelphia, Pennsylvania, United States of America; University of South Florida, United States of America

## Abstract

In this study, rat bone marrow stromal stem cells (BMSCs) were tracked after IV administration to rats with experimental stroke caused by middle cerebral artery occlusion (MCAO). In addition, the effects of BMSC treatment on blood cell composition, brain glia and sensorimotor behavior was studied and compared to that which occurred spontaneously during the normal recovery process after stroke. We found that the vast majority of radiolabeled or fluorescently labeled BMSCs traveled to and remained in peripheral organs (lungs, spleen, liver) 3 days after IV injection in the MCAO rat. Once in the circulation, BMSCs also produced rapid alterations in host blood cell composition, increasing both neutrophil and total white blood cell count by 6 hours post-injection. In contrast, few injected BMSCs traveled to the brain and almost none endured there long term. Nonetheless, BMSC treatment produced dramatic changes in the number and activation of brain astroglia and microglia, particularly in the region of the infarct. These cellular changes were correlated with a marked improvement in performance on tests of sensory and motor function as compared to the partial recovery of function seen in PBS-injected control rats. We conclude that the notable recovery in function observed after systemic administration of BMSCs to MCAO rats is likely due to the cellular changes in blood and/or brain cell number, activation state and their cytokine/growth factor products.

## Introduction

Currently, stroke is the leading cause of disability and the second leading cause of death worldwide. With the advent of anti-thrombosis treatment in the last decade [1], [2], the mortality rate associated with stroke has declined somewhat (12%). However, since only a small fraction of ischemic patients benefit from anti-thrombotic therapy, the overall incidence of stroke continues to climb, as does its enormous burden on society (for review, [3]. Therefore, developing new ways to treat stroke remains of utmost importance.

Stem cells, with their ability to both stimulate endogenous repair mechanisms and replace dying neurons offer great promise as a potential stroke therapy [4], [5]. Indeed, studies over the last decade have shown a marked improvement in brain structure and function following stem cell transplant, regardless of the route of cell delivery (intraparenchymal, intraventricular, intravenous; IV) [6], [7] or the type of stem cell administered (bone marrow, umbilical cord blood, ES cells) (for review, [8], [9]). Despite this progress, there remain fundamental gaps in our understanding regarding the fate of stem cells once transplanted and the mechanism of action underlying their beneficial effects.

In this study, we have employed imaging techniques to track the journey followed by radiolabeled or fluorescently labeled rat bone marrow stromal stem cells (BMSCs) after IV injection into rats with experimental stroke caused by middle cerebral artery occlusion (MCAO). In addition, the effects of BMSC treatment on blood cell composition, brain glia and sensorimotor behavior have been chronicled during an extended period following infarction and compared to those that occur during normal spontaneous recovery from stroke. We will show that while brain structure and function can partially recover from experimental stroke, IV administration of BMSCs greatly increases the extent of that recovery. The latter occurs despite the fact that the vast majority of BMSCs remain in the periphery after systemic injection into the MCAO rat where they produce changes in blood cell composition. Likewise, in the brain where few injected BMSCs endure long term, there are dramatic changes in the number and activation of astroglia and microglia in the region of the infarct that is correlated with an improvement in behavioral measures. We conclude that the sustained recovery in sensorimotor function observed in BMSC-treated MCAO rats is likely due to the changes in blood and/or brain cells and/or their cytokine/growth factor products.

## Materials and Methods

### Animals

#### Ethics Statement

All procedures in this study were carried out in accordance with the recommendations in the Guide for the Care and Use of Laboratory Animals of the National Institutes of Health. The protocol was approved by the IACUC Committee of the Thomas Jefferson University (Protocol Number: 457I). All surgery was performed under anesthesia, and all efforts were made to minimize suffering.

#### MCAO lesion

Adult, male, Sprague-Dawley rats weighing 275–350 grams were anesthetized using 60 mg/kg SQ ketamine hydrochloride, 10 mg/kg xylazine and 5 mg/kg acepromazine maleate. When necessary, depending on a reflex withdrawal response and breathing rate, maintenance doses of 10 mg/kg SQ ketamine hydrochloride, 1.5 mg/kg xylazine and 0.75 mg/kg acepromazine maleate were administered to maintain anesthesia. Atropine (0.054 mg/kg, SQ) was given during the procedure to decrease bronchial secretions and keep up the heart rate. Core body temperature was monitored with a rectal temperature probe, and maintained with a heating pad and/or a small fan to within 0.5°C.

Rats were shaved and artificial tears ointment was applied to the animal's eyes for protection and lubrication. The surgical site was scrubbed with Betadine, Chlorhexidine, and 70% ethyl alcohol. The animal was positioned in a prone position. A midline incision was made and the skull is thinned over the right parietal cortex (5 mm lateral and 1 mm posterior to Bregma) without injury to the dura mater with the burr hole. The laser Doppler was attached to the skull with dental cement. Cerebral blood flow was measured starting before, during and after ischemia (Blood Flow Meter, AD Instruments, Colorado Springs, CO). Then the animal was placed supine, and the MCAO performed. Briefly, the right common carotid (CCA) and external and internal carotid arteries (ECA, ICA) were exposed through a 2 cm midline incision. Using an operating microscope, the right CCA was ligated at the proximal end. Then the right ECA was ligated just distal to the branch of occipital artery. A microvascular clamp was used to temporarily occlude the pterygoplatine artery. The occipital artery was separated and cauterized. Then a 3-0 silk suture was used to place a slack knot around the right CCA between the first ligature and the bifurcation. Right ICA blood flow was then interrupted by clamping with a microvascular clip at its origin. A tiny opening on the right CCA was made between the first ligature and the slack knot. A silicone rubber-coated monofilament nylon suture (diameter with coating is 0.39 mm, Doccol) was then inserted into the lumen of the CCA through this opening. The slack silk suture was then constricted around the CCA over the nylon suture to avoid bleeding. The clamps were removed and the nylon suture was carefully advanced into the ICA until it obstructed the MCA (18 mm from the bifurcation of the CCA). Occlusion produced on average a 40–70% decline in blood flow as detected by laser Doppler. Two hours later, the constricted suture over the CCA was loosened and the filament removed for reperfusion of the brain. Sham operated animals were anesthetized and underwent surgery and brain microdissection as described above but without tying off of the arteries.

#### BMSC harvest and cell culture

Rat BMSCs were harvested from SD rats (Harlan) or from a transgenic rat cell line in which the human placental alkaline phosphatase (Alk phos) gene was placed under the control of the human ROSA26 regulatory element (Invitrogen). In brief, bone marrow cells were flushed from the shaft of the femur bone with PBS. Cells were then plated into medium containing 15% FCS, 1% nonessential amino acids, 1% glutamine, 1% pen-strep in DMEM. Cells were passaged 6–8 times before use in transplantation studies.

#### Cell labeling and transplantation procedures

For isotope labeling, cells were harvested in 200 uL of PBS. 1.1 mCi of In-111 oxine in 1 ml of PBS was added. Cells were incubated with tracer for 30 minutes at RT, followed by repeated rinses to remove unbound activity. BMSCs were isolated from the Alk phos transgenic rat line, allowing for the histochemical visualization of individually tagged BMSCs. Alk-phos-labeled BMSCs were harvested from culture using standard methods and tracked in vivo via histochemistry using the ELF® 97 Endogenous Phosphatase Detection Kit (Invitrogen) and visualized using an Olympus IX81 Image System. Labeled or unlabeled rat BMSCs were harvested from culture, centrifuged (1000 RPM) and the pellet re-suspended in sterilized PSB and injected 5×10^5^ cells in 0.5 mL of sterile saline into the rat tail vein after MCAO surgery.

#### Imaging Methods

To assess the functional deficits associated with infarction, we injected ^18F^-Fluorodeoxyglucose (FDG) intravenously into rats one day post-MCAO. Animals were fasted for 6 hours prior to each scan to eliminate any elevations in blood glucose at the time of the scan. In brief, the rat was anesthetized with Ketamine-Xylazine-Acepromizine mixture i.p. before the injection of tracer to avoid unnecessary uptake of tracer into the muscles due to the movement of the rat. We injected approximately 350 µCi of ^18^F-FDG intravenously through tail vein. The rat continued to be under the effect of anesthesia for another 90 minutes, during which time imaging procedures were performed. An Inveon pre-clinical PET scanner (Siemens, Knoxville, TN) was used for PET imaging. A 10 min PET acquisition was performed at 45 min from the time of injection of tracer in addition to the 10 min transmission imaging. A CT scan was performed in MicroCAT II CT scanner (Siemens, Knoxville, TN) after the PET imaging. After the scan, the animals were placed in quarantine in a biohazard hood. Rats were not returned to the animal colony until the radiation had completely decayed to background (24 hours for ^18^F).

For real-time tracking of injected BMSCs, rats were transported to the TJU small animal imaging facility. Usually 1–2 but no more than 4 animals were studied in any one session. To trace the fate of injected In-111 labeled BMSCs, cells were resuspended and slowly infused into the tail vein of a rat 24 hrs after MCAO surgery, followed by 0.5 ml of PBS “chaser” (final injected activity was approximately 570 uCi). CT-imaging was performed using a gamma camera. (each scan lasting up to 30 minutes) at 1 hour, 2 hours, 4 hours, 8 hours, 12 hours, 18 hours, 24 hours, 36 hours, and 48 hours after injection. Whole body images were obtained using a flat collimator and a pinhole collimator was used to capture brain images. The animals were anesthetized using 2% isoflurane for imaging and were allowed to recover between imaging time points any time the imaging interval was greater than 1 hour. The rats were monitored in the animal imaging facility until fully awake and recovered. Imaging was repeated for 3 sham-lesioned and 3 MCAO lesioned rats.

#### Blood Cell Analysis

To track changes in circulating blood cell populations following IV injection of BMSC's, host blood was collected from the tail vein before and 24 hr after MCAO and 6 and 24 hr after BMSC treatment. Blood was collected in 2 ml tubes spray-dried with 1.2–2.0 mg EDTA/1 mL of blood (Fischer Scientific Cat No. 22-040-101), samples were kept at 4°C and sent them to Bioreliance (Rockville, MD) for blood cell analysis. Briefly, cells were separated using an automated system that employs the ADVIA 120 Hematology analyzer to perform differential CBC assay using cytochemical light scatter and light absorption measurements and LIMS software analysis [10].

#### Behavioral Tests

To evaluate neurological function before and after stem cell transplant, rats were subjected to a battery of tests before and after MCAO and transplantation. Motor deficits were evaluated using a modified Neurological Severity Score (mNSS) [11] which includes fore and hind limb flexion; walking, righting, placing, balance, body resistance, paralysis). Using this scale, one point was given for the inability to perform a test. Consequently, the higher the score, the more severe is the motor deficit (maximum score = 18). In addition, motor function was assessed using a forelimb use asymmetry test [12]–[14], wherein rats were placed in a plexiglass cylinder and the number of attempts to climb the sidewalls with impaired (I), unimpaired (U) and both (B) limbs was quantified during a 10 min trial. The asymmetry index was calculated as [U/(U+I+B)] – [I/(U+I+B)] [12]–[14]. The higher the index, the greater is the asymmetry in use. We also used a corner test wherein rats were placed in a 30 degree corner and the percentage of turns to the right (unimpaired side) was calculated for 15 trials [12], [15]. Since normal rats turn 50% of the time towards the right and 50% toward the left side, the higher the score, the greater the MCAO-induced deficit. Behavior was assessed at regular intervals by an observer blinded to treatment status.

#### Immunocytochemistry

Transplanted rat brains were perfused with 4% cold (4°C) periodate-lysine-paraformaldehyde, sectioned at 30 µm on a freezing microtome and processed for immunocytochemistry using the immunofluorescence staining method described previously [16]. Cultures or sections through brain, spleen, liver and heart were stained with one or more of the following antibodies: mouse anti-human nuclear antigen (HNA) from Millipore at 1∶40; rabbit antibodies to GFAP, 1∶1000; monoclonal mouse antibodies to CD11, 1∶25. All secondary antibodies were Alexa Fluor antibodies from Invitrogen used at 1∶200–1∶300. Cultures or sections were analyzed using a Nikon-Scanalytics Image System or an Olympus IX81 Image System. After immunostaining, five regions of interest surrounding the infarct on the side of MCAO, and comparable regions in the contralateral and PBS control striatum, were quantified in 10 sections through each forebrain. Image J software (http://rsb.info.nih.gov/ij/) was used to quantify pixel intensity values and percent relative values determined. The data were analyzed using GraphPad Prism software with a non-paired Student's *t*-test.

#### TTC Staining method

Following sacrifice, fresh (unfixed) brain was removed from the skull, washed in iced PBS and placed in a brain mold. Coronal sections 1 mm in thickness were cut through the cerebrum and placed in 1% 2,3, 5 triphenyltetrazolium chloride (TTC; Sigma) for 30 min in a 3°C incubator. TTC solution was drained and sections were immersed in 4% formalin in 0.1 m PB at 4°C and stained as described previously [16].

#### Statistical Analysis

Data were statistically analyzed by one-way analysis of variance. When P<0.05, then the *F*-test was followed by the two-tailed Students t-test to compare the statistical significance between control and experimental groups (ANOVA with subsequent post-hoc analysis using Bonferroni correction). Statistical differences were considered significant only when the *P* value was<0.05.

## Results

### Assessing stroke and its effects

**Figure 1:**
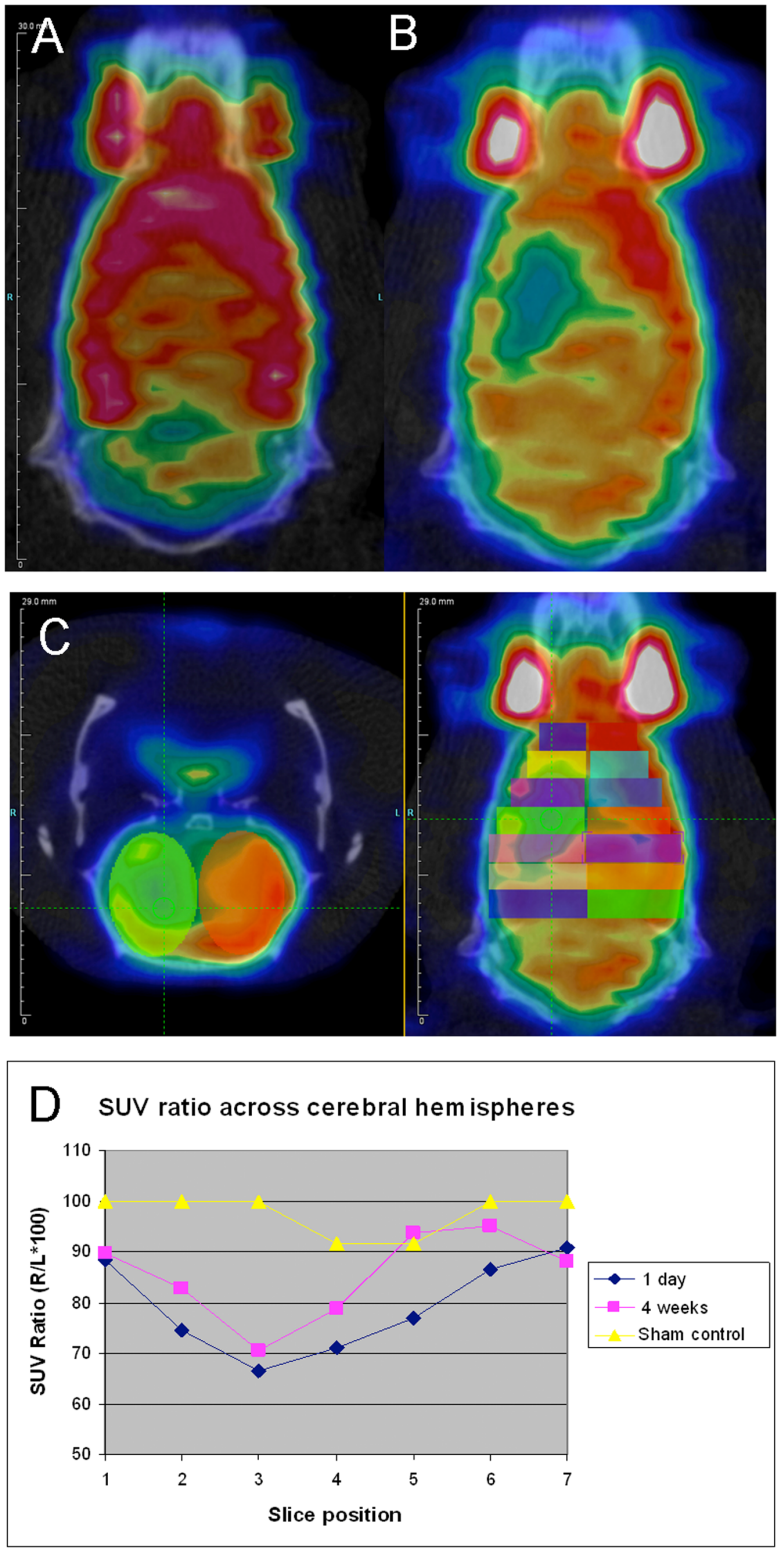
Analysis of infarct severity and extent on PET/CT imaging in representative sham and MCAO brains. A. Coronal view of a sham-operated animal showing normal symmetrical glucose uptake. B. One day post-MCAO, there is decreased uptake on the side of the infarct. C. Axial and coronal images showing placement of 2 mm thick ovoid regions of interest (ROIs) [symmetrical across the midline, arbitrary colors] across the cerebral hemispheres. D. Mean standardized uptake value (SUV) ratios (infarcted to contralateral side ROI) are plotted against slice position comparing infarcted and sham-operated control rat shown in A–C. The severity of the infarct is reflected both by the number of slices affected, and the degree to which activity is suppressed (relative to normal) in each slice. Over 4 weeks, there was a modest spontaneous improvement in FDG uptake, which correlated with the improvement seen in mNSS scores from 6 to 4 (not shown). Imaging was repeated for 3 sham-lesioned and 3 MCAO lesioned rats.

To assess functional change in the brain and body, fluorodeoxyglucose (^18^F) (FDG) utilization was imaged in rats for up to a month following experimental stroke and compared to sham-operated controls (n = 3). Reconstruction was performed with 3D-OSEM algorithm to get high resolution images of brain metabolism. A CT scan was performed after the PET imaging and image registration was used to align CT images and PET images. PET-CT fusion images were used to identify anatomical landmarks. Regions of interest (ROI) were drawn on the PET images to measure the amount of glucose metabolism in different parts of the brain. From these, we calculated the size of the infarct and the improvement in uptake at different time points (1, 4 weeks). We found that using FDG, one could image the region and functional loss after MCAO and even assess the improvement which occurs spontaneously over time (example provided in **Fig. 1**). Note that over a 4 week recovery period, slice 3, near the greatest area of infarct, spontaneously recovered only 4% while slices 2 and 4–6 showed greater recovery (8–22%) and slices 1 and 7 where there is collateral circulation from the anterior and posterior cerebral were less affected (10%) by occlusion of the MCA.

**Figure 2:**
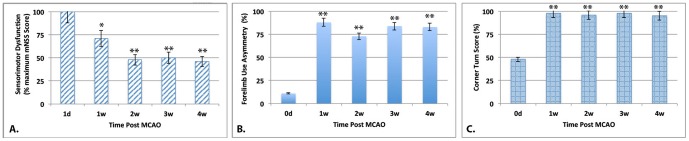
Spontaneous recovery of behavioral deficits after MCAO. Panel A represents sensorimotor loss as measured by the mNSS score. mNSS scores are normalized to a maximum of 18 on the mNSS scale (100% deficit). Note that following MCAO there was substantial spontaneous recovery in the mNSS score. N = 4–6, *p<0.01 and **p<0.001 compared to scores at 1 d post-MCAO. Panel B represents the rats' ability to climb the sidewall of a cylinder using the affected limb after MCAO. All rats were tested prior to MCAO and at 1, 2, 3 and 4 weeks after MCAO and the asymmetry index calculated (See Methods). Note that the decline in forelimb climbing remained even at 4 weeks post-MCAO. N = 4–6, ** p<0.001. Panel C represents the ability of the test animal to turn toward the unimpaired right side when placed in a corner. All rats are tested prior to MCAO, turning 50% of the time to the right and 50% of the time to the left. Values represent % of trials in which the rat turned toward the unimpaired side after MCAO. Note that following MCAO, this ability declined dramatically. ** p<0.001. In all 3 panels, the higher the value, the greater the deficit. (N = 4–8 ± SEM).

Importantly, the improvement reported above was accompanied by a substantial degree of spontaneous recovery in some but not all sensorimotor behaviors. In studies done to date on MCAO rats, the modified Neurological Severity Score (mNSS) has been the most widely used scale of behaviors to assess changes in neurological function [11]. As anticipated, after a major infarct in the brain, there was a significant decline in sensorimotor function on mNSS score (Mean score of 14/18) at 1 day post-MCAO (set to 100%) (N = 5–8). However, 1 week later, deficits were markedly reduced (27% improvement in the mNSS score; **Fig. 2A**) in infarcted rats receiving only palliative care, due particularly to improvements in gait, body resistance and spontaneous movement. By 2–4 weeks after injury, there was further recovery that plateaued when the mNSS score reached approximately 50% of the original 1 day post-MCAO score. In contrast, other behaviors, such as, use of the impaired forelimb in climbing (**Fig. 2B**), and ability to turn toward both left and right in the corner test (**Fig. 2C**), which relies on both sensory function of the vibrissae and motor function on the impaired side [12], [15], did not show substantial spontaneous recovery in function over the course of a month.

#### In vivo tracking of radioisotope-labeled BMSCs in MCAO rats

**Figure 3:**
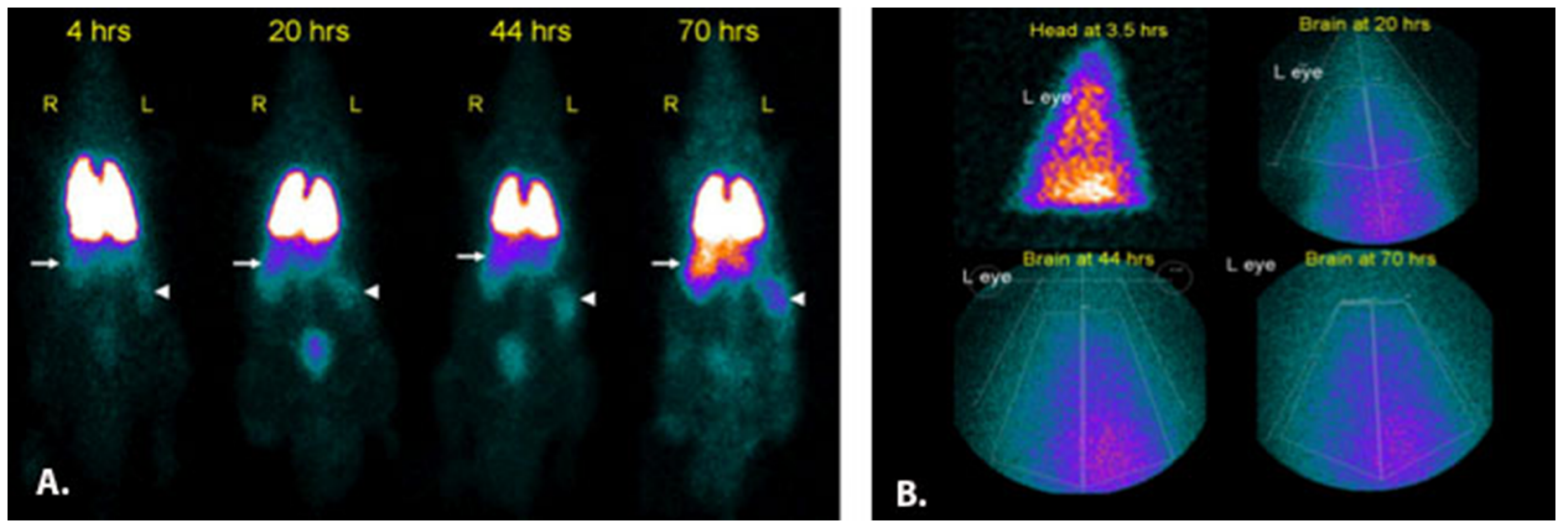
Whole body and brain images following IV injection of In-111 oxine–labeled BMSCs. (A) Images show persistent activity in the lungs, with relative activity in the liver (arrows) and spleen (arrowheads) increasing over time. Bladder activity is also seen. (B) Brain images at various times after injection. Larger magnification through a pinhole collimator with increasing proximity to the forebrain in later images (20 hrs, 44 hrs, 70 hrs) showed higher activity on the side of the infarct. Approximate position of left eye is marked in each image. (N = 4).

As little is yet known about the path that BMSCs travel after their injection into the bloodstream, we tracked the journey of radioisotope-labeled BMSCs in rats with moderate to severe stroke. To do so, BMSCs (8.1×10^6^ cells) were harvested and grown as described by us previously [16]. For isotope labeling, BMSCs were harvested in 200 uL of PBS. 1.1 mCi of In-111 oxine in 1 ml of PBS was added. Cells were incubated with tracer for 30 minutes at RT, followed by repeated rinses to remove unbound activity. Cells were re-suspended and slowly infused into the tail vein of a rat 24 hrs after MCAO surgery, followed by 0.5 ml of PBS “chaser” (final injected activity was approximately 570 uCi). Imaging was performed using a gamma camera. Whole body images were obtained using a flat collimator, at 4, 20, 44 and 70 hrs after injection, with 350,000 counts collected for each image. We found that the vast majority of IV-injected cells traveled to the lungs where they remained 3 days later. In addition, activity in the liver and spleen increased over time (**Fig. 3A**). Images of the brain were obtained using a pinhole collimator at 20, 44 and 70 hours, with 100,000 counts collected for each image. By 44 and 70 hours, higher brain activity was observed on the side ipsilateral to the infarct, suggesting that MSCs had in fact homed to the site of brain injury (**Fig. 3B**).

### Tracking transplanted alkaline phosphatase labeled BMSCs in MCAO rats

**Figure 4:**
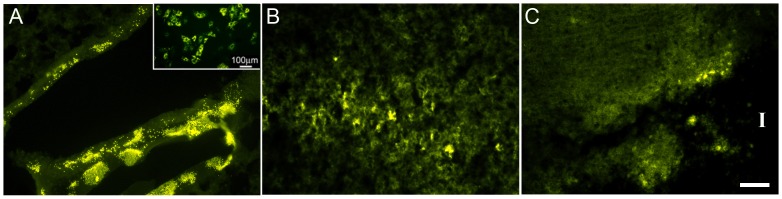
Alkaline phosphatase labeled rBMSCs in vitro and after IV administration into MCAO rats. Panel A.: Lung sections 3 days after transplantation; BMSCs show intense activity in the small airways. Note distinct green punctate Alk Phos histochemical staining. Inset: Human alk phos in BMSCs in culture. B. Brain sections from the same animal showing activity in the peri-infarcted tissue. C. Brain at 8 days after transplantation shows some activity at the edge of the infarct zone (“I” = infarct, with no intact tissue). (N = 3) Scale bars in A,C = 100 um; B = 50 um.

Although the radioisotope imaging technique is useful as a first level of investigation, resolution at the cellular level requires additional analysis. Therefore, we used a labeling technique which allows for histochemical visualization of individual Alk phos tagged BMSCs (Invitrogen). BMSCs (derived as described in Methods) robustly expressed Alk phos during cell expansion and differentiation in culture (**Fig. 4A** **inset**). After IV injection into the MCAO rat tail vein, Alk phos-tagged cells were readily detected in the lung (**Fig. 4A**) and in the brain at days **3** post-transplant (**Fig. 4B**). However, most labeled BMSCs in the brain had vanished by 8 days post-transplant although some remained at the edge of the infarcted region (**Fig. 4C**).

### Changes in host cells after BMSC treatment in MCAO rats: Analysis of Host Blood Cell Composition

**Figure 5:**
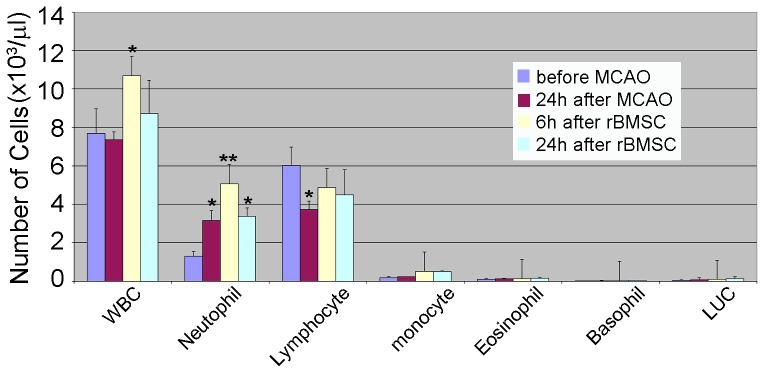
Analysis of blood composition before and after MCAO and rat BMSC treatment. Blood was collected from the tail vein before and 24 hr after MCAO and 6 and 24 hours after IV injection of BMSCs and quantitatively analyzed for counts of white blood cells (WBC), neutrophils, lymphocytes, monocytes, eosinophils, basophils and large unstained cells (LUC) by Bioreliance (Rockville, MD). (N = 3) *p<0.05 and **p<0.01.

Although many labs have examined the effects of BMSCs in the brain, no one has tracked their effects on host blood cells. To assess changes in blood cell populations following IV injection of BMSC's, the blood was collected from the tail vein before and 24 hr after MCAO and 6 and 24 hr after BMSC injection. We found that MCAO procedure alone produced an increase in neutrophils, a decrease in lymphocytes and no change in other cell types or the total white blood cell count (**Fig. 5**). However, by 6 hours after BMSC treatment, there was a further amplification in the number of neutrophils, resulting in an increase in the total while blood cell count despite the fact that lymphocytes remained unchanged from MCAO-induced levels (**Fig. 5**). Importantly, by 24 h hours after stem cell treatment, all cell counts reverted to pretreatment or post-MCAO levels.

### Analysis of BMSC Effects on the Brain

**Figure 6:**
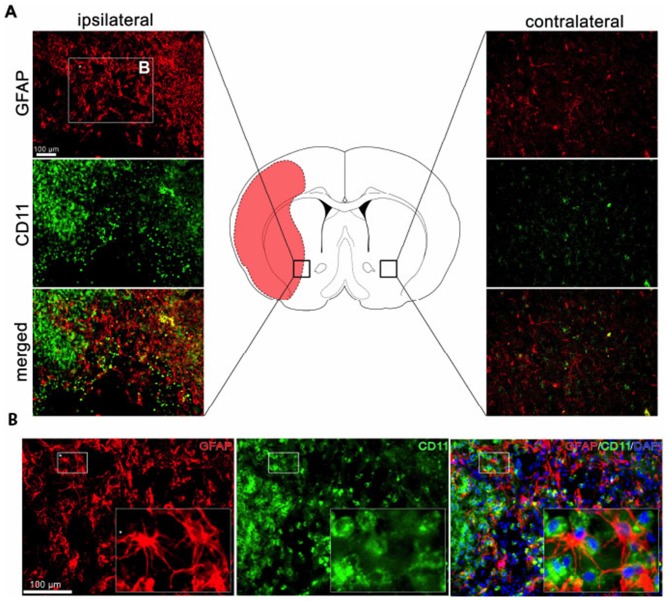
Immunocytochemical analysis of reactive glia and activated microglia in the region of the infarct 4 weeks after BMSC treatment in MCAO rats. Panel A. Representative section through the brain of MCAO rat (center) showing area of infarction (red). Area in rectangle on ipsilateral (left) or contralateral (right) sides of MCAO shown enlarged after staining with GFAP (reactive astrocytes) and CD11 (activated microglia) or merged images in side panels. Panel B. Area labeled “B” in rectangle of Panel A is shown at higher power after staining for GFAP or CD11; Insets: magnified small rectangles of B. Bars in A and B = 100 µm. (N = 4).

As shown previously [16]–[18], BMSC treatment produces significant changes in local brain cells beyond that which occurs due to MCAO. Thus, by 1 week (data not shown) and continuing at 4 weeks post-injection (**Fig. 6A**), rats administered BMSCs 24 hrs after MCAO exhibited a marked increase in the number of reactive GFAP^+^ astrocytes (412%±29% relative fluorescence intensity) and CD11^+^ microglia (487%±17% relative fluorescence intensity) in the area surrounding the infarct compared to the contralateral side (100%±9% relative fluorescence intensity) or PBS-treated MCAO rats (98%±12%). In addition, glia and microglial cells in the stem cell-treated rats exhibited an activated morphology, shown in **Fig. 6B** insets, at 4 weeks, long after their counterparts in untreated MCAO rats had returned to the quiescent state (**Fig. S1**), as noted previously [16].

### Recovery of function after BMSCs treatment in MCAO rats

**Figure 7:**
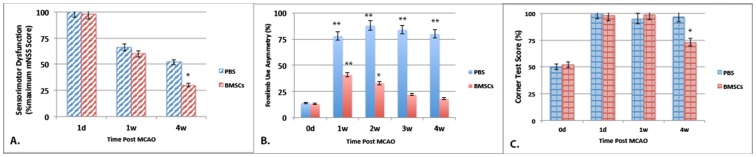
Correlation of BMSC treatment with a significant improvement in behavior over time. All behavioral assessments as described in Fig. legend 2. Panel A. Sensorimotor behavior as assessed on the mNSS scale 1 or 4 weeks after PBS or BMSC treatment (compared to mNSS score at 1 day post-MCAO set to 100%). Panel B. Climbing ability as assessed on the forelimb asymmetry test at 1, 2, 3 and 4 weeks after PBS or BMSC treatment. When compared to pre-MCAO performance on day 0, PBS groups wer significantly different at all treatment times while those treated with BMSCs differed at 1 and 2 weeks post-treatment but normalized towards control at later time points. Importantly, forelimb asymmetry was significantly different (p<0.001) between BMSC and PBS treatment groups at all 4 treatment times. Panel C. Corner test evaluated at 1 day, 1 and 4 weeks after PBS or BMSC treatment (compared to pre-MCAO performance on day 0). N = 6, *p<0.01 and **p<0.001.

Importantly, changes seen in the brain after BMSC treatment were correlated with functional recovery of sensorimotor behaviors when compared to control rats (PBS-treated). Thus, stem cell treatment resulted in a significant improvement in neurologic function (ie. mNSS score) (**Fig. 7A**) over the partial recovery seen spontaneously after MCAO (**Fig. 2A**). In addition, BMSC treatment significantly improved other long-lasting measures of sensorimotor function, such as cylinder climbing capacity and left corner turning ability, both of which did not show signs of spontaneous recovery (**Figs. 7B,C**). In all of these cases, BMSC-induced recovery in behavioral deficits did not reach significance until 4 weeks after treatment was initiated.

## Discussion

Numerous studies have chronicled the remarkable ability of systemic BMSCs to produce recovery of brain structure and function after experimental stroke in rats [9], [16], [19]–[23]. However, few if any studies have adequately assessed the degree to which these indices improve spontaneously over time without interventional treatment. In addition, relatively few studies have actually tracked the journey taken by stem cells after their systemic administration and documented their effects in blood and brain during the recovery period [24]–[26]. The current study demonstrates that the brain is indeed capable of considerable spontaneous recovery in structure and function after experimental stroke but that systemic BMSC treatment, which affects both blood and brain cell composition, provides added benefit, improving all indices examined, particularly at later post-recovery times.

Until now, the role of spontaneous recovery of function after MCAO has not been well-investigated. The present study however found that even after a large MCAO-induced infarction, there was a partial but significant restoration in brain glucose function. In fact, the high demand for energy in the brain is almost exclusively provided by the oxidative metabolism of glucose. Importantly, brain regions at greater distances from the infarct showed considerable (22%) spontaneous recovery of glucose utilization, while contrastingly, areas of greatest infarct showed much less recovery of glucose function (4%). These findings suggest that most rescue in glucose function occurs in the penumbra region surrounding the infarct, as has been noted previously [27]–[29]. Moreover, regions with greater collateral blood circulation from the anterior and posterior cerebral arteries are able to sustain a drop in blood flow without significant decline in glucose utilization.

In examining how functional changes in glucose uptake correlated with changes in sensorimotor function, a somewhat different profile emerged, depending on the behavioral assessment. We found that during the first 1–2 weeks after MCAO, there was a substantial degree of recovery in the mNSS score, which assesses a wide range of motor and sensory skills. The recovery stemmed mainly from improvements in gait, body resistance and spontaneous activity in MCAO rats. However, by, 2 weeks post-MCAO, this recovery plateaued, with no further improvement at later times. In contrast to the mNSS score, other sensorimotor tests affected by MCAO remained unchanged one month later. Thus, there was no spontaneous recovery in the ability to use the affected limb to climb the sidewalls of a cylinder and virtually no improvement in the rats' ability to turn towards the impaired side in the corner test one month after MCAO. While both of the latter tests assess motor function, the corner test also depends upon sensory stimulation of the vibrissae to induce turning behavior [12], [15]. Thus, in judging the success of new therapies for stroke, caution must be exercised as some behaviors partially improve spontaneously over time after MCAO, while others do not.

Additionally, our study examined whether therapeutic treatment with systemic BMSCs could ameliorate the deficits in brain structure and function caused by MCAO, enhancing recovery beyond that which occurs spontaneously. Despite the wide array of studies describing stem cells and their effects after experimental stroke in the rat [9], [16], [19], [20], [22], [23], [30], little is known about the route that BMSCs take in the body and brain when administered directly into the peripheral circulation. In our imaging experiments, we found that most IV-injected BMSCs remained in the periphery, lodging in the lung, the first capillary bed reached after tail vein injection and in the liver and spleen. More importantly, when cells were labeled with Indium-111 and scanned 3 days later or when Alk phos-labeled cells were imaged by fluorescence 8 days after transplantation, relatively few BMSCs were found in the brain, suggesting that the vast majority of cells either did not successfully cross the infarct-damaged blood-brain-barrier [31] or perished soon after their arrival there. Similarly, in a recent study tracking radiolabeled cells after intra-arterial infusion into the external carotid artery, the majority of cells that made it to the brain disappeared within 24 hours [24]. Although the brain is thought to be immune-privileged, it is likely that BMSCs were eliminated via immune rejection mechanisms [32]. Interestingly, recent studies using GFP-labeled BMSCs injected directly into brain parenchyma contrastingly found labeled cells in the brain many weeks after transplantation [33], [34], suggesting that the absence of BMSCs in the brain in our study may be initiated by rejection-related mechanisms in the periphery.

Thus, in this regard, it was particularly interesting to find that the IV injection of BMSCs into the circulation resulted in a rapid (within 6 hours of treatment) alteration in the composition of blood. In particular, we noted a significant increase in host neutrophils, the white blood cells which are the usual first responders to inflammation [35]. This correlates well with changes in blood cytokines (ie. VEGF, MIP, MMP2) noted previously after BMSC treatment by us and others [16], [35]–[37]. Although we did not look for the enhanced presence of neutrophils in the brain, the fact that these cells readily migrate across blood vessels suggests that the increase in circulating neutrophils observed after systemic BMSCs could readily result in greater numbers of neutrophils or their products in the brain [31], [35]. Also, in regions where there has been a significant breakdown in the BBB integrity due to the infarct, white blood cells could gain easy access to the brain, causing cytokines and chemokines to be released into the parenchyma [31], [32].

How these stem cell-induced changes modify the well-documented inflammatory response which occurs in the host brain after experimental stroke [18], [35], [38], [39] is only now being explored. We found that BMSC treatment in MCAO rats indeed results in a marked up-regulation in the number and in the activation state of local CD11^+^ microglia and GFAP^+^ astrocytes, particularly in the penumbra surrounding the infarct. These changes persist even at one month after MCAO, that is, several weeks after the MCAO-induced glial response has returned to normal [16], [40] and the infarcted region has been walled off by astrocytes in the ischemic brain [16]. Thus, BMSC-induced cellular alterations in the host glia, combined with the reported increase in vasculogenesis [41]–[44], suggests that stem cells and/or their products play a pivotal role in partitioning off damage, safe-guarding tissue integrity and/or possibly promoting regeneration in the brain after experimental stroke.

Of significance, these BMSC-induced cellular changes in brain were associated with a substantial improvement in all behavioral indices examined, including those that had partially recovered spontaneously after MCAO. Thus, at 4 weeks post-MCAO, performance on the mNSS, climbing and corner turning tests were significantly improved in the BMSC treatment group compared to PBS controls. Since 4 weeks was the latest time point examined, in the future, it will be important to extend the time course of these studies to determine whether even greater recovery of function is possible at later time points or after multiple treatments with BMSCs. A similar lag in functional recovery in electrophysiology, FMRI and behavioral parameters has indeed been reported previously [45]. Whether these delayed changes in brain structure and function owe to direct effects from the increase in blood neutrophils or from the few BMSCs found in the brain or instead from secondary processes initiated by their cell products (cytokines/chemokines/growth factors) remains to be elucidated.

## Supporting Information

Figure S1 Immunocytochemical analysis of reactive GFAP+ glia and activated CD11+ microglia in the striatum on the side of the infarct at 1 (A, B) and 4 (C,D) weeks after MCAO in PBS-infused rats. Note that in control (IV infusion of PBS) rats, there is considerable microglial activation and some astroglial reactivity in the areas adjacent to the infarct as a result of MCAO. However, by 4 weeks, much of this activation/reactivity due to MCAO had receded. Bar = 100 µm. (N = 4).
(TIF)

## References

[1] Vendrame M, Gemma C, De Mesquita D, Collier L, Bickford PC, et al (2005) Anti-inflammatory effects of human cord blood cells in a rat model of stroke. Stem cells and development 14: 595–604 Available: http://www.ncbi.nlm.nih.gov/pubmed/16305344. Accessed 9 December 2012. 16305344 10.1089/scd.2005.14.595

[2] Lalancette-Hebert M, Phaneuf D, Soucy G, Weng YC, Kriz J (2009) Live imaging of Toll-like receptor 2 response in cerebral ischaemia reveals a role of olfactory bulb microglia as modulators of inflammation. Brain Available: 10.1093/brain/awn345. 10.1093/brain/awn345 19153151

[3] Eissa A, Krass I, Bajorek BV (2012) Optimizing the management of acute ischaemic stroke: a review of the utilization of intravenous recombinant tissue plasminogen activator (tPA). Journal of clinical pharmacy and therapeutics 37: 620–629 Available: http://www.ncbi.nlm.nih.gov/pubmed/22708668. Accessed 9 December 2012. 22708668 10.1111/j.1365-2710.2012.01366.x

[4] Ramos-Cabrer P, Justicia C, Wiedermann D, Hoehn M (2010) Stem cell mediation of functional recovery after stroke in the rat. PloS one 5: e12779 Available: http://www.pubmedcentral.nih.gov/articlerender.fcgi?artid=2943902&tool=pmcentrez&rendertype=abstract. Accessed 9 December 2012. 20877642 10.1371/journal.pone.0012779 PMC2943902

[5] De Feo D, Merlini A, Laterza C, Martino G (2012) Neural stem cell transplantation in central nervous system disorders: from cell replacement to neuroprotection. Current opinion in neurology 25: 322–333 Available: http://www.ncbi.nlm.nih.gov/pubmed/22547103. Accessed 9 December 2012. 22547103 10.1097/WCO.0b013e328352ec45

[6] Li L, Jiang Q, Ding G, Zhang L, Zhang ZG, et al (2010) Effects of administration route on migration and distribution of neural progenitor cells transplanted into rats with focal cerebral ischemia, an MRI study. Journal of cerebral blood flow and metabolism: official journal of the International Society of Cerebral Blood Flow and Metabolism 30: 653–662 Available: http://www.pubmedcentral.nih.gov/articlerender.fcgi?artid=2844252&tool=pmcentrez&rendertype=abstract. Accessed 9 December 2012. 10.1038/jcbfm.2009.238 PMC2844252 19888287

[7] Zhang L, Li Y, Romanko M, Kramer BC, Gosiewska A, et al (2012) Different routes of administration of human umbilical tissue-derived cells improve functional recovery in the rat after focal cerebral ischemia. Brain research 1489: 104–112 Available: http://www.ncbi.nlm.nih.gov/pubmed/23063717. Accessed 11 December 2012. 23063717 10.1016/j.brainres.2012.10.017

[8] Zhang ZG, Chopp M (2009) Neurorestorative therapies for stroke: underlying mechanisms and translation to the clinic. Lancet neurology 8: 491–500 Available: http://www.pubmedcentral.nih.gov/articlerender.fcgi?artid=2727708&tool=pmcentrez&rendertype=abstract. Accessed 18 November 2012. 19375666 10.1016/S1474-4422(09)70061-4 PMC2727708

[9] Sanberg PR, Eve DJ, Metcalf C, Borlongan CV (2012) Advantages and challenges of alternative sources of adult-derived stem cells for brain repair in stroke. Progress in brain research 201: 99–117 Available: http://www.ncbi.nlm.nih.gov/pubmed/23186712. Accessed 9 December 2012. 23186712 10.1016/B978-0-444-59544-7.00006-8

[10] Aulesa C, Mainar I, Prieto M, Cobos N, Galimany R (2003) Use of the Advia 120 hematology analyzer in the differential cytologic analysis of biological fluids (cerebrospinal, peritoneal, pleural, pericardial, synovial, and others). Laboratory hematology: official publication of the International Society for Laboratory Hematology 9: 214–224 Available: http://www.ncbi.nlm.nih.gov/pubmed/14649464. Accessed 24 January 2013. 14649464

[11] Sughrue ME, Mocco J, Komotar RJ, Mehra A, D'Ambrosio AL, et al (2006) An improved test of neurological dysfunction following transient focal cerebral ischemia in rats. Journal of neuroscience methods 151: 83–89 Available: http://www.ncbi.nlm.nih.gov/pubmed/16476486. Accessed 10 December 2012. 16476486 10.1016/j.jneumeth.2005.04.023

[12] Hua Y, Schallert T, Keep RF, Wu J, Hoff JT, et al (2002) Behavioral tests after intracerebral hemorrhage in the rat. Stroke; a journal of cerebral circulation 33: 2478–2484 Available: http://www.ncbi.nlm.nih.gov/pubmed/12364741. Accessed 9 December 2012. 10.1161/01.str.0000032302.91894.0f 12364741

[13] Schallert T, Fleming SM, Leasure JL, Tillerson JL, Bland ST (2000) CNS plasticity and assessment of forelimb sensorimotor outcome in unilateral rat models of stroke, cortical ablation, parkinsonism and spinal cord injury. Neuropharmacology 39: 777–787 Available: http://www.ncbi.nlm.nih.gov/pubmed/10699444. Accessed 26 April 2011. 10699444 10.1016/s0028-3908(00)00005-8

[14] Schallert T (2006) Behavioral tests for preclinical intervention assessment. NeuroRx: the journal of the American Society for Experimental NeuroTherapeutics 3: 497–504 Available: http://www.ncbi.nlm.nih.gov/pubmed/17012064. Accessed 9 December 2012. 17012064 10.1016/j.nurx.2006.08.001 PMC3593401

[15] Zhang L, Schallert T, Zhang ZG, Jiang Q, Arniego P, et al (2002) A test for detecting long-term sensorimotor dysfunction in the mouse after focal cerebral ischemia. Journal of neuroscience methods 117: 207–214 Available: http://www.ncbi.nlm.nih.gov/pubmed/12100987. Accessed 1 November 2012. 12100987 10.1016/s0165-0270(02)00114-0

[16] Yang M, Wei X, Li J, Heine LA, Rosenwasser R, et al (2010) Changes in host blood factors and brain glia accompanying the functional recovery after systemic administration of bone marrow stem cells in ischemic stroke rats. Cell transplantation 19: 1073–1084 Available: http://www.ncbi.nlm.nih.gov/pubmed/20412636. Accessed 10 December 2012. 20412636 10.3727/096368910X503415

[17] Lalancette-Hebert M, Gowing G, simard A, Weng YC, Kriz J (2007) Selective ablation of proliferating microglial cells exacerbates ischemic injury in the brain. J Neurosci 27: 2596–2605. 17344397 10.1523/JNEUROSCI.5360-06.2007 PMC6672496

[18] Kriz J (2006) Inflammation in ischemic brain injury: timing is important. Crit Rev Neurobiol 18: 145–157. 17725517 10.1615/critrevneurobiol.v18.i1-2.150

[19] Caplan AI (2007) Adult mesenchymal stem cells for tissue engineering versus regenerative medicine. Journal of cellular physiology 213: 341–347 Available: http://www.ncbi.nlm.nih.gov/pubmed/17620285. Accessed 3 November 2012. 17620285 10.1002/jcp.21200

[20] Hess DC, Borlongan C V (2008) Cell-based therapy in ischemic stroke. Expert review of neurotherapeutics 8: 1193–1201 Available: http://www.pubmedcentral.nih.gov/articlerender.fcgi?artid=2753686&tool=pmcentrez&rendertype=abstract. Accessed 9 December 2012. 18671663 10.1586/14737175.8.8.1193 PMC2753686

[21] Li WY, Choi YJ, Lee PH, Huh K, Kang YM, et al (2008) Mesenchymal stem cells for ischemic stroke: changes in effects after ex vivo culturing. Cell transplantation 17: 1045–1059 Available: http://www.ncbi.nlm.nih.gov/pubmed/19177841. Accessed 9 December 2012. 19177841 10.3727/096368908786991551

[22] Li Y, Chopp M (2009) Marrow stromal cell transplantation in stroke and traumatic brain injury. Neuroscience letters 456: 120–123 Available: http://www.pubmedcentral.nih.gov/articlerender.fcgi?artid=3359793&tool=pmcentrez&rendertype=abstract. Accessed 9 December 2012. 19429146 10.1016/j.neulet.2008.03.096 PMC3359793

[23] Gutiérrez-Fernández M, Rodríguez-Frutos B, Alvarez-Grech J, Vallejo-Cremades MT, Expósito-Alcaide M, et al (2011) Functional recovery after hematic administration of allogenic mesenchymal stem cells in acute ischemic stroke in rats. Neuroscience 175: 394–405 Available: http://www.ncbi.nlm.nih.gov/pubmed/21144885. Accessed 9 December 2012. 21144885 10.1016/j.neuroscience.2010.11.054

[24] Mitkari B, Kerkelä E, Nystedt J, Korhonen M, Mikkonen V, et al (2012) Intra-arterial infusion of human bone marrow-derived mesenchymal stem cells results in transient localization in the brain after cerebral ischemia in rats. Experimental neurology 239C: 158–162 Available: http://www.ncbi.nlm.nih.gov/pubmed/23059455. Accessed 9 December 2012. 10.1016/j.expneurol.2012.09.018 23059455

[25] Detante O, Moisan A, Dimastromatteo J, Richard M-J, Riou L, et al (2009) Intravenous administration of 99mTc-HMPAO-labeled human mesenchymal stem cells after stroke: in vivo imaging and biodistribution. Cell transplantation 18: 1369–1379 Available: http://www.ncbi.nlm.nih.gov/pubmed/19849895. Accessed 22 January 2013. 19849895 10.3727/096368909X474230

[26] Steiner B, Roch M, Holtkamp N, Kurtz A (2012) Systemically administered human bone marrow-derived mesenchymal stem home into peripheral organs but do not induce neuroprotective effects in the MCAo-mouse model for cerebral ischemia. Neuroscience letters 513: 25–30 Available: http://www.ncbi.nlm.nih.gov/pubmed/22342911. Accessed 22 January 2013. 22342911 10.1016/j.neulet.2012.01.078

[27] Astrup J, Siesjö BK, Symon L (1981) Thresholds in cerebral ischemia - the ischemic penumbra. Stroke; a journal of cerebral circulation 12: 723–725 Available: http://www.ncbi.nlm.nih.gov/pubmed/6272455. Accessed 9 December 2012. 10.1161/01.str.12.6.723 6272455

[28] Heiss WD (1992) Experimental evidence of ischemic thresholds and functional recovery. Stroke; a journal of cerebral circulation 23: 1668–1672 Available: http://www.ncbi.nlm.nih.gov/pubmed/1440719. Accessed 9 December 2012. 10.1161/01.str.23.11.1668 1440719

[29] Symon L (2007) The Ischemic Penumbra. 1st Editio. Donnan A. G, Baron J-C, Davis SM, Sharp FRS, editors New York: Informa Healthcare.

[30] Li WY, Choi YJ, Lee PH, Huh K, Kang YM, et al (2008) Mesenchymal stem cells for ischemic stroke: changes in effects after ex vivo culturing. Cell transplantation 17: 1045–1059 Available: http://www.ncbi.nlm.nih.gov/pubmed/19177841. Accessed 9 December 2012. 19177841 10.3727/096368908786991551

[31] Takeshita Y, Ransohoff RM (2012) Inflammatory cell trafficking across the blood-brain barrier: chemokine regulation and in vitro models. Immunological reviews 248: 228–239 Available: http://www.ncbi.nlm.nih.gov/pubmed/22725965. Accessed 9 December 2012. 22725965 10.1111/j.1600-065X.2012.01127.x PMC3383666

[32] Miller DW (1999) Immunobiology of the blood-brain barrier. Journal of neurovirology 5: 570–578 Available: http://www.ncbi.nlm.nih.gov/pubmed/10602398. Accessed 9 December 2012. 10602398 10.3109/13550289909021286

[33] Shichinohe H, Kuroda S, Lee J-B, Nishimura G, Yano S, et al (2004) In vivo tracking of bone marrow stromal cells transplanted into mice cerebral infarct by fluorescence optical imaging. Brain research Brain research protocols 13: 166–175 Available: http://www.ncbi.nlm.nih.gov/pubmed/15296854. Accessed 9 December 2012. 15296854 10.1016/j.brainresprot.2004.04.004

[34] Miyamoto M, Kuroda S, Zhao S, Magota K, Shichinohe H, et al (2012) Bone Marrow Stromal Cell Transplantation Enhances Recovery of Local Glucose Metabolism After Cerebral Infarction in Rats: A Serial 18F-FDG PET Study. Journal of nuclear medicine: official publication, Society of Nuclear Medicine Available: http://www.ncbi.nlm.nih.gov/pubmed/23204494. Accessed 9 December 2012. 10.2967/jnumed.112.109017 23204494

[35] Jin R, Yang G, Li G (2010) Inflammatory mechanisms in ischemic stroke: role of inflammatory cells. Journal of leukocyte biology 87: 779–789 Available: http://www.pubmedcentral.nih.gov/articlerender.fcgi?artid=2858674&tool=pmcentrez&rendertype=abstract. Accessed 13 November 2012. 20130219 10.1189/jlb.1109766 PMC2858674

[36] Deng YB, Ye WB, Hu ZZ, Yan Y, Wang Y, et al (2010) Intravenously administered BMSCs reduce neuronal apoptosis and promote neuronal proliferation through the release of VEGF after stroke in rats. Neurological research 32: 148–156 Available: http://www.ncbi.nlm.nih.gov/pubmed/19473555. Accessed 9 December 2012. 19473555 10.1179/174313209X414434

[37] Zhao Y, Li Z, Wang R, Wei J, Li G, et al (2010) Angiopoietin 1 counteracts vascular endothelial growth factor-induced blood-brain barrier permeability and alleviates ischemic injury in the early stages of transient focal cerebral ischemia in rats. Neurological research 32: 748–755 Available: http://www.ncbi.nlm.nih.gov/pubmed/19660197. Accessed 9 December 2012. 19660197 10.1179/016164109X12445616596562

[38] Lalancette-Hébert M, Gowing G, Simard A, Weng YC, Kriz J (2007) Selective ablation of proliferating microglial cells exacerbates ischemic injury in the brain. The Journal of neuroscience: the official journal of the Society for Neuroscience 27: 2596–2605 Available: http://www.ncbi.nlm.nih.gov/pubmed/17344397. Accessed 9 December 2012. 17344397 10.1523/JNEUROSCI.5360-06.2007 PMC6672496

[39] Kriz J, Lalancette-Hébert M (2009) Inflammation, plasticity and real-time imaging after cerebral ischemia. Acta neuropathologica 117: 497–509 Available: http://www.ncbi.nlm.nih.gov/pubmed/19225790. Accessed 9 December 2012. 19225790 10.1007/s00401-009-0496-1

[40] Pekney M, Wilhemsson U, Bogestal YR, Pekna M (2007) The Role of Astrocytes and Complement System in Neural Pasticity. Int Rev Neurobiol 82: 95–111. 17678957 10.1016/S0074-7742(07)82005-8

[41] Chen J, Zhang ZG, Li Y, Wang L, Xu YX, et al (2003) Intravenous administration of human bone marrow stromal cells induces angiogenesis in the ischemic boundary zone after stroke in rats. Circulation research 92: 692–699 Available: http://www.ncbi.nlm.nih.gov/pubmed/12609969. Accessed 9 December 2012. 12609969 10.1161/01.RES.0000063425.51108.8D

[42] Taguchi A, Soma T, Tanaka H, Kanda T, Nishimura H, et al (2004) Administration of CD34+ cells after stroke enhances neurogenesis via angiogenesis in a mouse model. J Clin Invest 114: 330–338. 15286799 10.1172/JCI20622 PMC484977

[43] Jiang Q, Zhang ZG, Ding GL, Zhang L, Ewing JR, et al (2005) Investigation of neural progenitor cell induced angiogenesis after embolic stroke in rat using MRI. NeuroImage 28: 698–707 Available: http://www.ncbi.nlm.nih.gov/pubmed/16112879. Accessed 9 December 2012. 16112879 10.1016/j.neuroimage.2005.06.063

[44] Shyu WC, Lin SZ, Chiang MF, Su CY, Li H (2006) Intracerebral peripheral blood stem cell (CD34+) implantation induces neuroplasticity by enhancing beta1 integrin-mediated angiogenesis in chronic stroke rats. J Neurosci 26: 3444–3453. 16571751 10.1523/JNEUROSCI.5165-05.2006 PMC6673856

[45] Ramos-Cabrer P, Justicia C, Wiedermann D, Hoehn M (2010) Stem cell mediation of functional recovery after stroke in the rat. PloS one 5: e12779 Available: http://www.pubmedcentral.nih.gov/articlerender.fcgi?artid=2943902&tool=pmcentrez&rendertype=abstract. Accessed 9 December 2012. 20877642 10.1371/journal.pone.0012779 PMC2943902

