# Supplementary figures and images for: Tracking Transplanted Bone Marrow Stem Cells and Their Effects in the Rat MCAO Stroke Model
Source: PLoS One. 2013 Mar 29;8(3):e60049. doi: 10.1371/journal.pone.0060049 (PMC3612030; doi:10.1371/journal.pone.0060049)

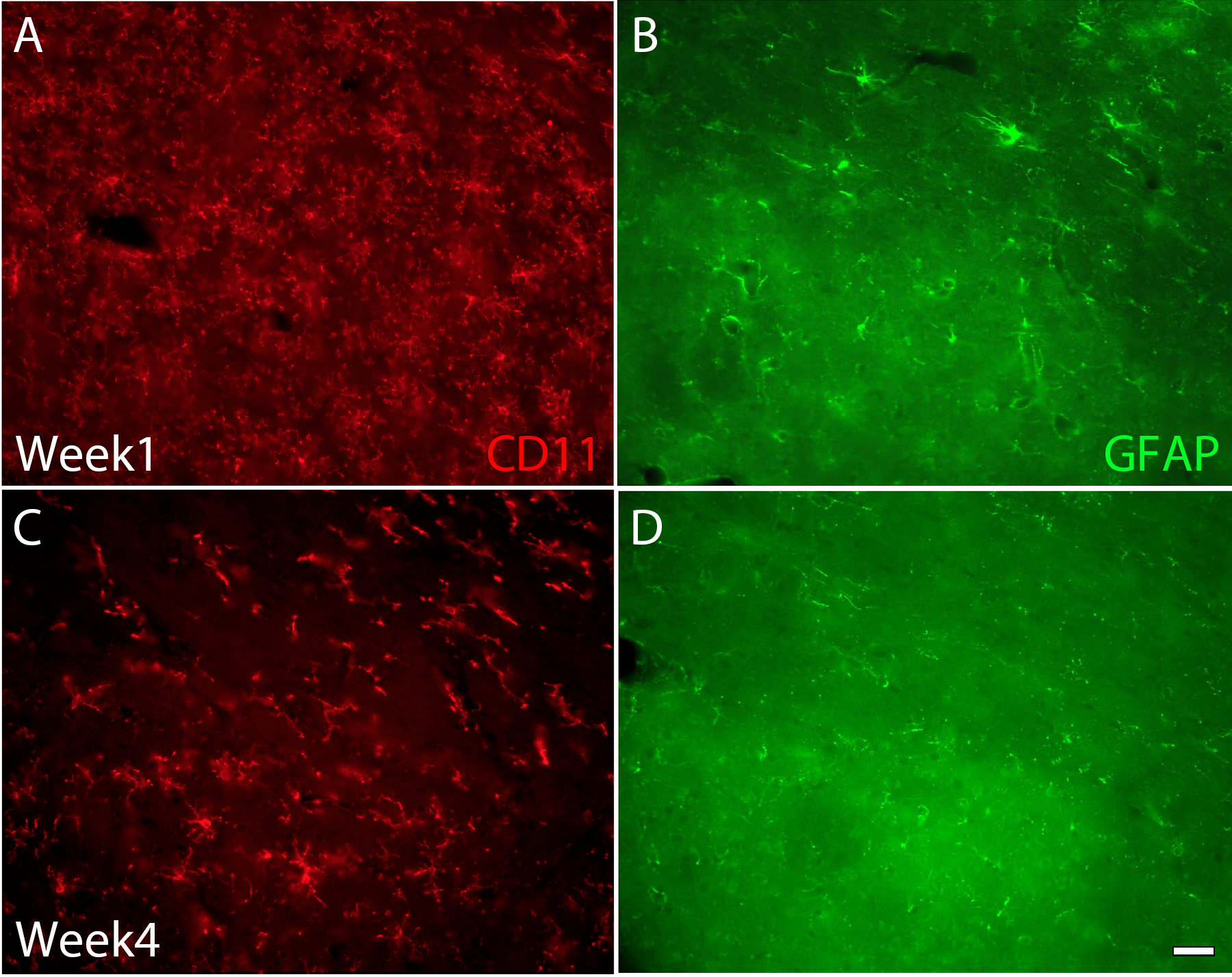

Supplement: Figure S1 — Immunocytochemical analysis of reactive GFAP+ glia and activated CD11+ microglia in the striatum on the side of the infarct at 1 (A, B) and 4 (C,D) weeks after MCAO in PBS-infused rats. Note that in control (IV infusion of PBS) rats, there is considerable microglial activation and some astroglial reactivity in the areas adjacent to the infarct as a result of MCAO. However, by 4 weeks, much of this activation/reactivity due to MCAO had receded. Bar = 100 µm. (N = 4). (TIF) [file pone.0060049.s001.tif]
